# Case Report: Mevalonate kinase deficiency: an underdiagnosed cause of ischemic stroke—characterization of a novel genetic variant

**DOI:** 10.3389/fimmu.2025.1651819

**Published:** 2025-10-03

**Authors:** Lyna-Nour Hamidi, Jack Christopher Drda, Meriem Belhocine, Hannah-Laure Elfassy, Stéphanie Ducharme-Bénard, Maxime Chayer-Lanthier, Bushra Sultana, Sylvain Lanthier

**Affiliations:** ^1^ Department of Genetics, McGill University, Montreal, QC, Canada; ^2^ Department of Microbiology, University of Pittsburgh, Pittsburgh, PA, United States; ^3^ Department of Internal Medicine, Hôpital du Sacré-Coeur de Montréal, Montreal, QC, Canada; ^4^ Faculty of Medicine, Université de Montréal, Montreal, QC, Canada; ^5^ Department of Immuno-allergology, Hôpital du Sacré-Coeur de Montréal, Montréal, QC, Canada; ^6^ Faculty of Medicine, University of Sherbrooke, Sherbrooke, QC, Canada; ^7^ Department of Neurology, Hôpital du Sacré-Coeur de Montréal, Montreal, QC, Canada; ^8^ Research Centre, Centre intégré de santé et de services sociaux du Nord de l’île de Montréal, Montréal, QC, Canada

**Keywords:** auto-inflammation, ischemic stroke, mevalonate kinase deficiency, neuroinflammation, vasculopathy

## Abstract

Mevalonate kinase deficiency (MKD) is an inherited autoinflammatory syndrome resulting from impaired isoprenoid biosynthesis due to biallelic mevalonate kinase (*MVK*) mutations. This metabolic defect leads to dysregulated innate immunity, particularly excessive interleukin-1β release. While typically presenting in childhood with periodic fevers, expanding evidence links MKD to heterogeneous adult phenotypes with immune-mediated end-organ damage. We report an adult male presenting with leg pain and finger cyanosis followed by acute ischemic stroke, macular rash, and lymphadenopathies. He exhibited classical markers of innate immune activation, including persistent elevation of C-reactive protein. Genetic testing identified compound heterozygosity for the known *MVK* pathogenic variant c.1129G>A (V377I) and a novel missense variant, c.1049A>C (Q350P). Structural modeling of Q350P revealed disruption of the GHMP kinase domain, predicted to destabilize mevalonate kinase conformation and impair its function. The measurement of mevalonate kinase activity in lymphocytes was at 55% (normal >60%). Interleukin-1β blockade with canakinumab was initiated, and the blood markers of inflammation normalized, further supporting a central role for innate immune dysregulation. This case highlights a novel *MVK* missense variant (Q350P) with subnormal mevalonate kinase activity. The patient’s compound heterozygous state with partially preserved mevalonate kinase activity may explain the attenuated systemic features and the delayed clinical onset. Remarkably, ischemic stroke was part of the initial presentation, suggesting that mevalonate kinase deficiency can manifest primarily through thrombo-inflammatory complications in adulthood, even in the absence of recurrent febrile episodes. This expands the phenotypic spectrum of MKD and underscores the need to consider adult-onset autoinflammatory syndromes in the differential diagnosis of cryptogenic ischemic strokes with markers of systemic inflammation. It also supports the utility of cytokine-targeted therapies in such contexts.

## Background

Autoimmune diseases are defined by adaptive immune system dysfunction, with lymphocytes attacking self-antigens. In contrast, autoinflammatory diseases arise from antigen-independent hyperactivation of innate immunity. Mevalonate kinase deficiency (MKD) is a monogenic, autosomal recessive, autoinflammatory disease resulting from biallelic pathogenic variants in the mevalonate kinase (*MVK)* gene. The downstream effects include defective protein prenylation, inflammasome activation, and hypersecretion of pro-inflammatory cytokines such as interleukin-1 (IL-1) (1). MKD encompasses a spectrum of phenotypes partially correlated with the *MVK* variants and residual enzymatic activity (2). Severely affected patients typically retain undetectable to 0.5% mevalonate kinase activity, often presenting with constitutive mevalonic aciduria, prominent neurological and ophthalmic involvement, and chronic systemic inflammation (3). Classically, milder forms preserve 1% to 20%–28% enzymatic activity (4) and exhibit recurrent febrile episodes with aphtosis, abdominal pain, diarrhea, arthromyalgia, painful lymphadenopathies, maculopapular rash, and other manifestations (3, 5). Attacks last for 3–7 days and are often triggered by fatigue, physical exertion, infections, or vaccination. In mild MKD, urinary mevalonic acid is usually detectable only during attacks. Atypical MKD phenotypes are increasingly reported, and the spectrum of MKD phenotypes continues to expand within the medical literature (3). We recently reported the first case of MKD-related inflammation causing ischemic stroke (IS) (6).

## Methods

We now report the second known MKD patient with an inflammatory flare complicated by IS. This patient harbors the common pathogenic c.1129G>A (V377I) variant in addition to a novel c.1049A>C (Q350P) variant. Mevalonate kinase activity in lymphocytes was measured to confirm the pathogenicity of the novel variant. By computational modeling, we analyzed the structural consequences of the Q350P substitution on mevalonate kinase. The amino acid sequence of mevalonate kinase (2R3V) was obtained from the Protein Data Bank (7) and encoded into AlphaFold3 (8). The predicted structure of mevalonate kinase was visualized in PyMOL (https://www.pymol.org) to identify the structural GHMP domain and compared to the known X-ray crystallography of mevalonate kinase (9). The glutamine 350 residue was localized, and all amino acid residues within 5 Å were identified. The amino acid sequence of mevalonate kinase was modified and substituted with a proline residue at position 350. The resulting sequence was encoded into AlphaFold3 and visualized with PyMOL. The tertiary structure of mevalonate kinase was compared between the wild-type and the novel Q350P variant.

## Results

### Case report

A 58-year-old man presented with acute hemiparesis. His past medical history included active cigarette and cannabis smoking, well-controlled hypertension, dyslipidemia, presbycusis, traumatic amputation of the left 5th toe, perforated intestinal ulcer operated on around age 35, and meniscal surgery at age 40. Being adopted, his parental background was unknown, except for being of French-Canadian descent. His four children and one granddaughter were healthy. One grandson underwent bilateral leg amputation in the context of toxic shock at age 4 years.

**Figure 1:**
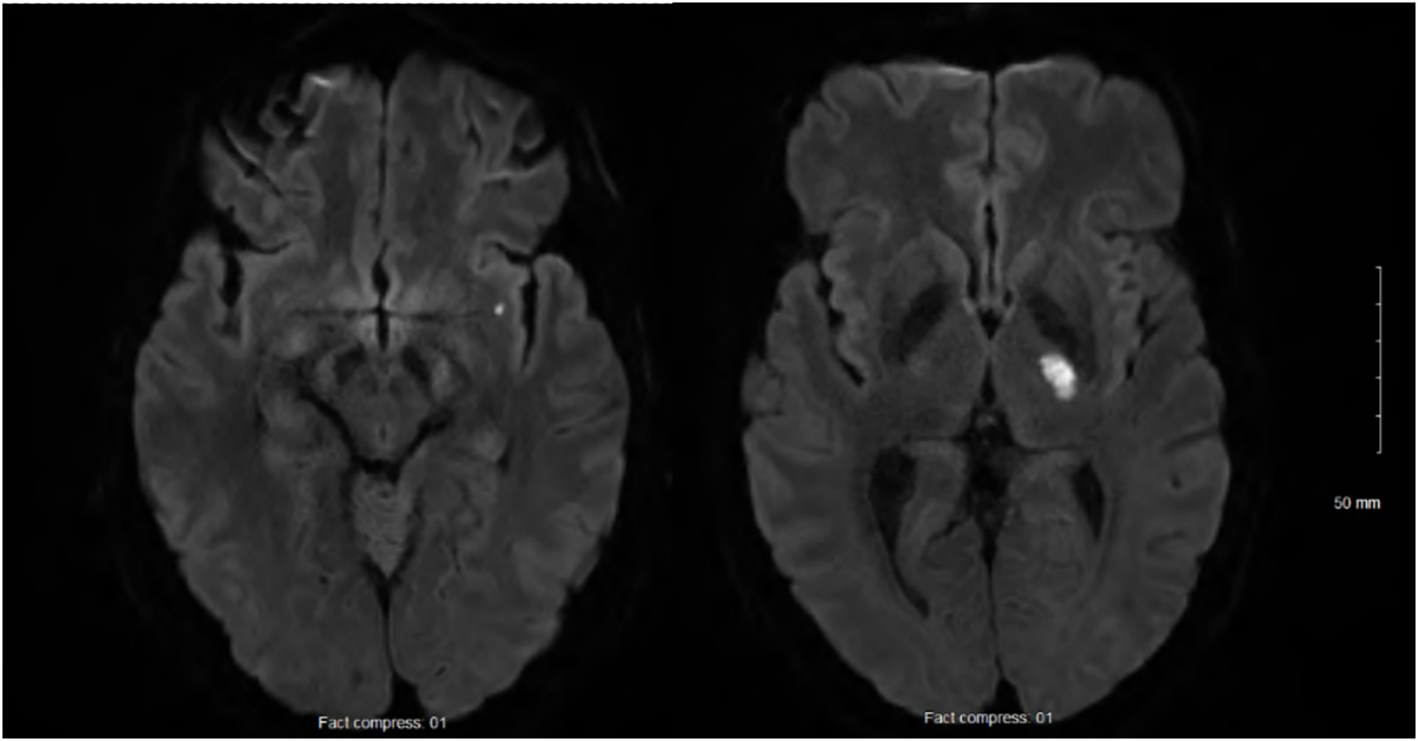
Acute small-sized infarcts of the left internal capsule and sub-insula, and no chronic infarcts, leucoaraiosis, or microbleeds.

At 24h after walking outside on a cold winter day, he developed pain in both legs and finger cyanosis on his right hand, which he attributed to frostbite despite wearing appropriate clothing. He had never experienced Raynaud’s phenomenon. At 2 weeks later, as he was improving, he woke up with recurrent leg pain and right-sided weakness. The physical exam showed normal vital signs and temperature, right hemiparesis, hypoesthesia limited to the medial aspect of the dorsum of the right foot and the first two toes, and normal myotatic reflexes. The last three fingers of the right hand were lightly cyanotic, combined with a subungual hematoma on the 5th. There was a slight orthostatic bluish discoloration of the lower limbs, worse on the right. There was no livedo racemosa and no dermatological manifestations. Brain magnetic resonance imaging (MRI) confirmed two acute small-sized infarcts consistent with small artery occlusion, without chronic ischemic or hemorrhagic changes (**Figure 1**). Antiplatelet therapy was initiated, consisting of aspirin (combined with clopidogrel for 3 weeks). Over the following days, he developed a thoraco-abdominal macular rash, which was initially thought to possibly result from allergy. However, the skin prick tests and drug challenges including aspirin and clopidogrel were negative, and the rash resolved spontaneously while the antiplatelet agents were continued.

Computed tomography (CT) angiography revealed atherosclerosis with 50% stenosis of the left proximal vertebral artery, no involvement upstream of the infarctions except for minimal pre-petrosal infiltration of the left internal carotid artery, and mild to moderate atherosclerosis of the abdominal aorta, superior mesenteric artery, and proximal lower limbs. There was no distal embolism or evidence of vasculitis. The results of transthoracic and transesophageal echocardiography, as well as cardiac monitoring by telemetry and 24-h Holter monitor, were unremarkable.

**Figure 2:**
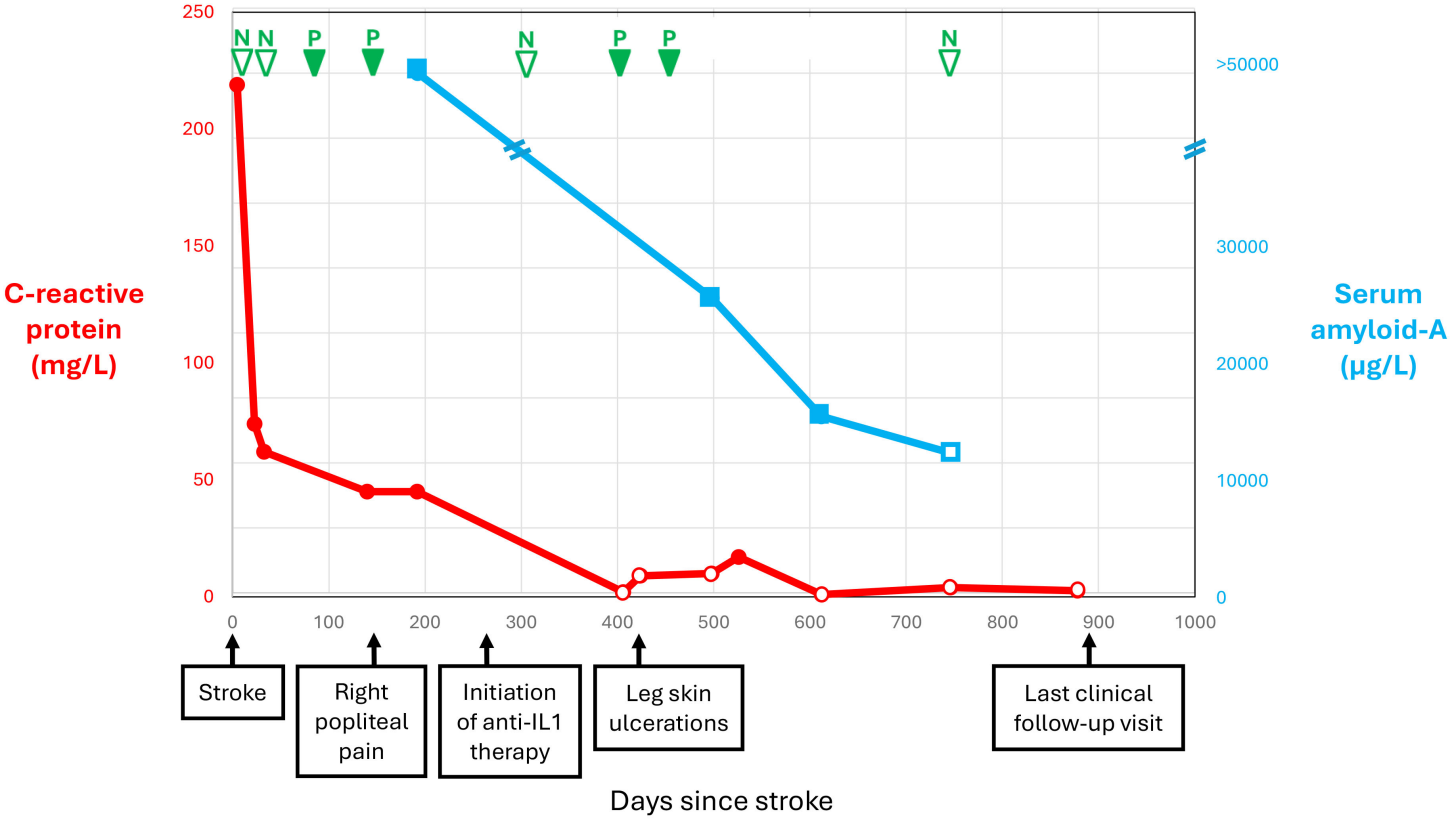
Selected blood test results and clinical manifestations over time since stroke.

**Figure 2** shows selected blood test results at presentation and during follow-up. The blood tests documented leukocytosis (19 × 10^9^/L), normocytic anemia (hemoglobin 135 g/L), thrombocytosis (668 × 10^9^/L), and increased C-reactive protein (CRP; 219 mg/L), ferritin (768.9 ug/L), fibrinogen (9.72 g/L), and interleukin-6 (23.8 pg/L). The results of coagulation tests were normal, including antiphospholipid antibodies. Autoimmune workup was unremarkable, including antinuclear antibodies, extractable nuclear antibodies, antineutrophil cytoplasmic antibodies, rheumatoid factor, complement, and direct Coombs test. Plasma protein electrophoresis was consistent with inflammation. Immunoelectrophoresis found monoclonal gammopathy immunoglobulin G-kappa (7.7 g/L) with an increased free light chain ratio (2.09). The immunoglobulin D and E levels were normal. Variants in the *janus kinase 2*, *calreticulin*, and *adenosine deaminase 2* genes were not detected. Fluorescent aerolysin assay was negative. Cryoglobulin and cold agglutinin were not detected, as was cryofibrinogen initially. Hepatitis B, hepatitis C, and human immunodeficiency virus tests were negative. Thoraco-abdominal-pelvic CT revealed mild emphysema. Whole-body fluorodeoxyglucose positron emission tomography showed nonspecific hypermetabolism of the axillary and inguinal lymph nodes, with no evidence of cancer or vasculitis. Lower limb pain and focal numbness were further investigated. Lumbosacral spine MRI and venous Doppler ultrasound were both negative. An electromyogram showed an axonal injury of the left peroneal nerve and mild changes to the left tibial nerve. Inflammation of unknown etiology and a negative infectious workup prompted the next-generation sequencing of 336 genes, which is part of a primary immunodeficiency panel. While the genetic test results were pending, the patient was improving overall.

At 5 months following IS, he experienced acute right popliteal pain extending to the ankle and exacerbated by movement, unassociated with evidence of lower limb ischemia or venous thrombosis at clinical examination and ultrasound. Repeat blood tests confirmed persistent inflammation, now including cryofibrinogen detected on four of five measurements over 11 months (**Figure 2**). The complement 4 levels had become low (0.10 and 0.13 g/L), while the complement 3 levels and cryoglobulin measurement remained normal. A repeat electromyogram showed additional axonal injury to the right tibial, peroneal, and bilateral sural nerves, appearing as a confluent sensorimotor polyneuropathy limited to the lower limbs. Gene testing came back positive for *MVK* trans compound heterozygosity, combining the c.1129G>A (V377I) pathogenic mutation and a novel c.1049A>C (Q350P) variant, and found no other autoinflammatory gene variants or modifiers. The patient’s grandson tested heterozygote for the V377I variant. Serum amyloid-A was >50,000 ng/mL. Urinary mevalonic acid was normal in between flares. Unfortunately, it could not be measured during a flare. Coverage approval was requested from Quebec province’s health insurance plan for anti-interleukin-1β monoclonal antibody treatment with canakinumab.

Canakinumab coverage was approved at 9 months following IS. Canakinumab was initiated at a dose of 150 mg every 4 weeks (**Figure 2**). The pain subsided almost immediately after treatment initiation. After 5 months of canakinumab treatment, the patient presented with leg skin ulcers with a necrotic center, unprovoked by cold exposure. Punch biopsy documented non-specific fibrinoid necrosis and granulation tissue, but no vasculitic changes, and the clinical presentation was consistent with pyoderma gangrenosum. The ulcerations regressed with very potent topical corticosteroids combined with high-dose prednisone and dapsone for a few months. Over time, the CRP and serum amyloid-A levels normalized, and cryofibrinogen became undetectable (**Figure 2**). The blood tests confirmed monoclonal gammopathy. Whole-body low-dose CT scan as well as bone marrow biopsy did not reveal multiple myeloma. A repeat EMG after 11 months of treatment remained stable with a confluent sensorimotor distal polyneuropathy, but no additional nerve injury. The residual mevalonate kinase activity in lymphocytes was measured. Given the strong response to canakinumab, we expected residual activity in the classic range of 1% to 20%–28% for MKD but found 55%, which remains less than the normal values (>60%). We are now at 21 months of treatment with canakinumab, and the patient has remained asymptomatic.

### Structural analysis by computational modeling of the Q350P substitution

**Figure 3:**
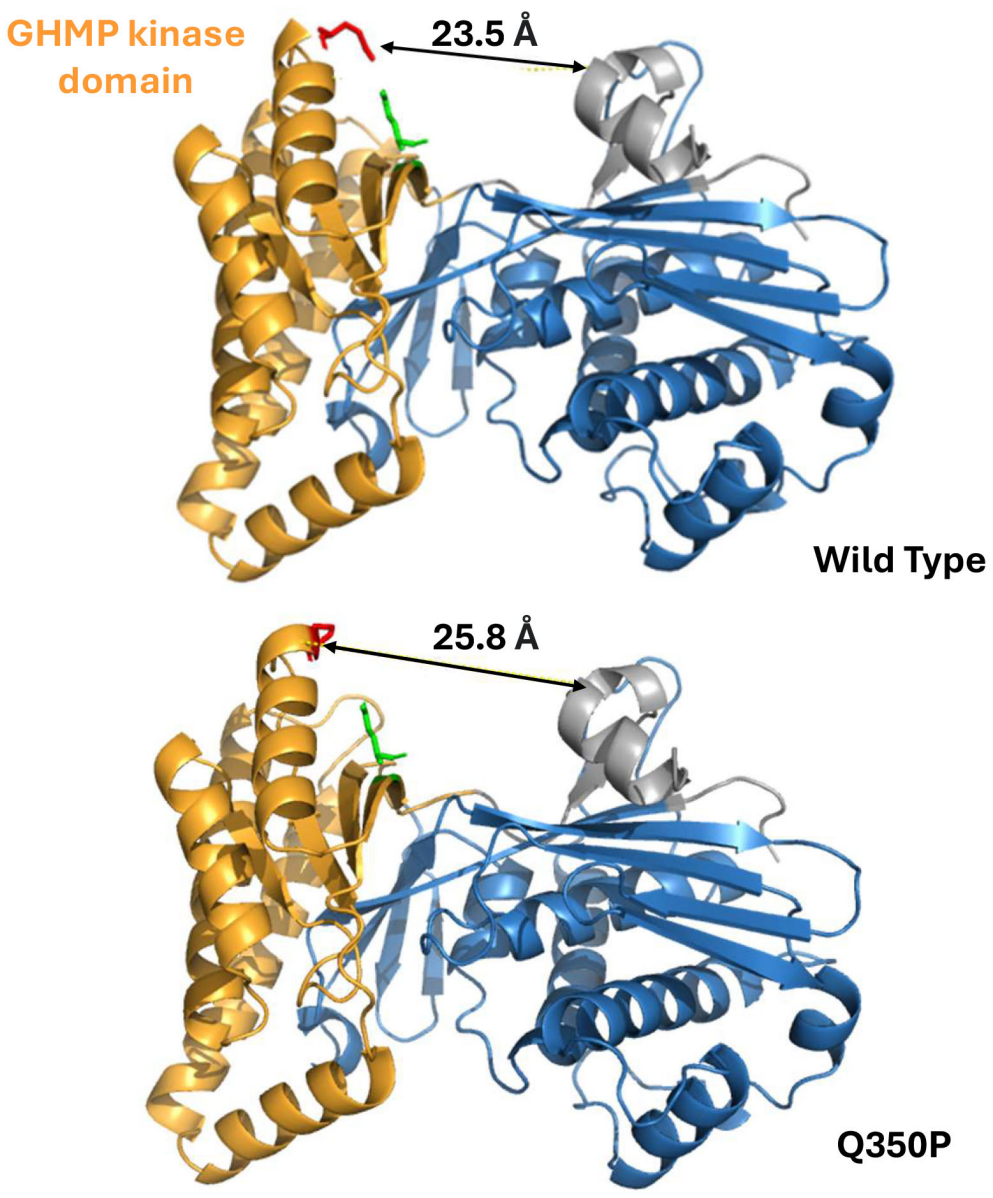
Mevalonate kinase computational analysis of conformational change resulting from the novel c.1049A>C (Q350P) variant. (Top) Wild type. (Bottom) Q350P. The novel variant abolished a polar-charge interaction within the mevalonate kinase tertiary structure, leading to an approximately 2.3 Å expansion between the functional domains. Substitution of a proline residue also added one more helical turn in the GHMP domain.

The predicted distance between the GHMP (galactokinase, homoserine kinase, mevalonate kinase, and phosphomevalonate kinase family) C-terminal and the N-terminal domain was noticeably expanded in the Q350P variant compared to the wild-type mevalonate kinase, indicating a probable disruption in mevalonate kinase activity (**Figure 3**). Substitution of a glutamine residue with proline eliminated a polar-charge interaction between Q350 and E369, respectively. This leads to an expansion between the two functional domains of mevalonate kinase, the region where mevalonate binds to the active site for phosphorylation. The substitution of a proline residue at residue 350 is adjacent to another proline residue at 351. Two sequential proline residues increased steric hindrance, leading to an additional helical turn in the Q350P variant. Taken together, the Q350P variant of mevalonate kinase likely remains pathogenic due to its alteration of tertiary structure and elimination of stabilizing electrostatic interactions.

## Discussion

The patient displayed trans compound heterozygosity for the known pathogenic V377I variant and the novel Q350P variant in the *MVK* gene. The Q350P substitution is located within a highly conserved kinase domain during evolution C-terminal GHMP kinase domain formed of residues 227-374 (4). The GHMP domain, named after four metabolic enzymes participating in essential phosphorylation reactions (with M standing for mevalonate kinase), is necessary for ATP and substrate binding (10). The Q350P substitution involves replacing a polar amino acid (glutamine) with a non-polar amino acid (proline). Proline substitutions are known to alter the protein tertiary structure and thermal stability due to their cyclic structure and lack of amide hydrogen to participate in peptide backbone hydrogen bonding (11). By computational modeling, we demonstrated that Q350P substitution alters mevalonate kinase tertiary structure, especially in its highly conserved kinase domain, which is essential for its function (**Figure 3**). The Q350P variant is therefore likely pathogenic, expanding the spectrum of MKD disorders.

Consistent with MKD, this patient presented with macular rash, lymphadenopathies, and increased inflammatory blood markers, spontaneous clinical improvement occurred, and laboratory evidence of inflammation persisted until anti-interleukin-1β antibody treatment was initiated (**Figure 2**). The favorable clinical response to canakinumab supports a central role for innate immune dysregulation, possibly through subthreshold activation of the nucleotide-binding domain leucine-rich repeat receptor family pyrin domain containing 3 (NLRP3) inflammasome and endothelial dysfunction in the presence of partial mevalonate kinase activity (12). Like this patient, 20% of MKD patients have normal immunoglobulin D levels, and recent studies have shown relatively low sensitivity and specificity of hyperimmunoglobulinemia D for MKD (3). His presentation in adulthood and the absence of prior repeated inflammatory crises are atypical for MKD, probably reflecting his residual mevalonate kinase activity, which ranges between normal and typically pathogenic levels. Atypical forms of MKD with prominent skin, liver, inflammatory bowel, and cardiorespiratory disease or amyloidosis were described in association with variable levels of residual mevalonate activity, resulting in delayed diagnosis (3). Adults with biallelic pathogenic mutations presenting late in life without recurrent inflammation or with no-disease phenotypes were also previously reported (13). Indeed approximately 300 MKD cases have been reported worldwide. MKD seems considerably rarer than expected from population studies of allele frequencies (https://gnomad.broadinstitute.org), suggesting an underestimation of its prevalence due to a delay between symptom onset and diagnosis (14), lack of awareness in the medical community, and milder presentations reflecting low penetrance (15).

**Figure 4:**
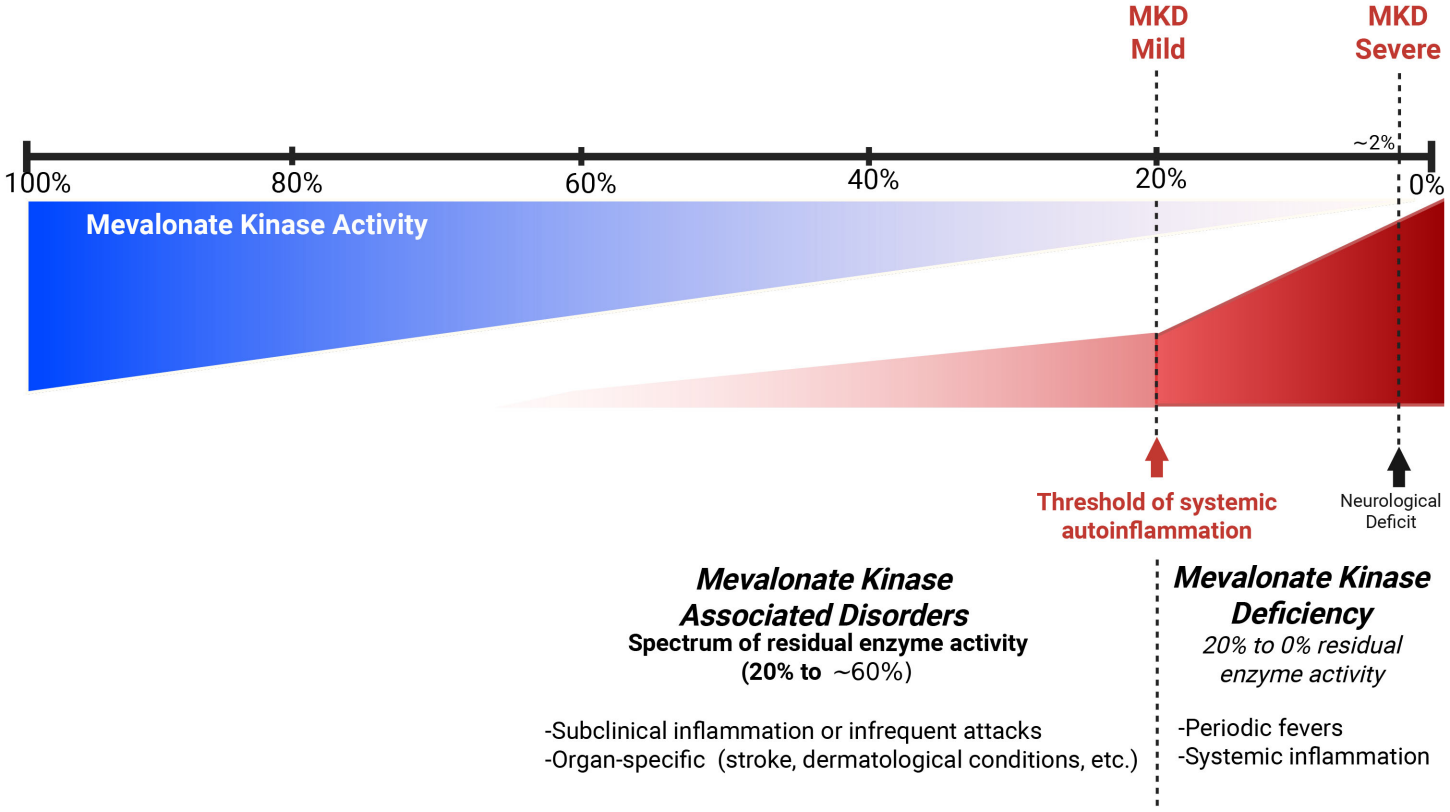
Hypothetical model of spectrum of mevalonate kinase associated disorders.

Residual enzyme activity was found to be 55% in this patient. Typically, MKD patients present a residual mevalonate kinase activity of anywhere between undetectable levels and as high as 20%–28% (5). In the recent years, mevalonate kinase-associated disorders have been increasingly recognized as a spectrum of disease rather than a limited set of distinct phenotypes (**Figure 4**) (3). Since MKD is known to be a very rare and underdiagnosed condition, we do not hold enough data to discard patients with subnormal but higher than 20%–28% enzyme activity. Heterozygous carriers for MKD (V377I) have been reported to be symptomatic and respond well to canakinumab (16). The patient that we report exhibited a strong response to anti-IL1 canakinumab, both clinically and through blood markers of inflammation, supporting an autoinflammatory disease diagnosis and a mevalonate kinase spectrum disorder. We hypothesize that patients with 20%–60% of residual mevalonate kinase activity may have an attenuated phenotype. This could lead to reduced intensity or frequency of systemic auto-inflammatory episodes and periodic fever and late occurrence of auto-inflammatory-related events, such as the case presented here. Mouse models show a spectrum of protein prenylation and inflammation based on the residual enzymatic activity, which should be between 50% and 75% (4). Symptomatic individuals with intermediate enzymatic activity of known autoinflammatory genes are reported in other autoinflammatory diseases, including familial Mediterranean fever and adenosine deaminase deficiency 2 (17, 18). Our patient shows compound heterozygosity with a known pathogenic variant (V377I) and a novel missense Q350P variant. The pathogenicity of this novel variant remains to be fully understood, but we hypothesize that the change in the tertiary structure of the protein and its thermal stability support its pathogenicity (4, 15).

The investigation found no common cause of IS. Lacunar IS due to arteriolosclerosis is unlikely in the absence of chronic ischemic or hemorrhagic changes on brain MRI. The investigation documented inflammation, but positron emission tomography and skin biopsy showed no evidence of vasculitis. Moreover, the patient’s favorable outcome in the absence of immunosuppressant treatment does not support vasculitis. We believe that our patient had an IS due to MKD-related inflammation and small artery occlusion. Abnormal activation of sophisticated interlinks between innate immunity and hemostasis is involved in thromboembolic complications of autoinflammatory diseases (19). We hypothesize that our patient’s risk factors for arteriosclerosis, including cigarette and cannabis smoking and dyslipidemia, and actual atherosclerotic plaques contributed to the systemic prothrombotic environment of thromboinflammation. Unfortunately, the biomarkers of endothelial dysfunction (e.g., vascular cell adhesion molecule-1, intercellular adhesion molecule 1) were not measured. The presence of cryofibrinogen and monoclonal gammopathy raises doubt about the IS etiology, and there is a potential for overlapping of different causes. Whereas essential cryofibrinogenemia is frequently asymptomatic, symptomatic patients typically present in the fifth decade with cold intolerance, skin ulcers affecting exposed surfaces, livedo, and thrombosis (20, 21).However, in the patient that we report here, there were no dermatological manifestations or livedo, and the blood tests did not find cryofibrinogen at IS presentation and on a subsequent measurement (**Figure 2**). Cryofibrinogen was variably detected from 3 months post-stroke until canakinumab treatment. Skin biopsy revealed nonspecific changes, without plugging of deep and superficial blood vessels by cryofibrinogen-containing thrombi (20, 21). Cryofibrinogenemia in this patient likely resulted from autoinflammation rather than being the cause of IS and skin ulcers (22). While skin biopsy findings could be consistent with PG, occurring in association with the documented monoclonal gammopathy of unknown significance, it can also represent a manifestation of MKD or its treatment with anti-IL1 agents. Histopathology of skin biopsies in MKD is varied and under-reported to date (23). This patient had monoclonal gammopathy but no evidence of multiple myeloma. Monoclonal gammopathy may increase the risk of thrombosis. However, monoclonal gammopathy of thrombotic significance is generally not associated with inflammation, and the absence of recurrent thromboembolic events when no treatment targeting this condition has been initiated does not support this diagnosis (24).

Our patient is the second reported case with IS attributed to MKD. The only other patient with IS secondary to MKD was reported from our center (6). Unlike the current patient, this first case suffered from recurrent systemic inflammatory attacks from a young age. In both cases, the IS resulted from arteriolar occlusion during an attack. IS due to small artery occlusion is likewise reported in other autoinflammatory diseases, including familial Mediterranean fever and adenosine deaminase 2 deficiency (17, 18). To our knowledge, no other MKD patients presenting with IS in adulthood have been previously reported. A survey of 50 patients with MKD identified a single asymptomatic adult. This 40-year-old patient was a homozygous carrier of the pathogenic variant c.1129G>A (V377I) and had a mevalonate kinase activity level of 9.7% (25). This supports the finding that pre-symptomatic individuals may develop clinical manifestations of MKD late in life.

## Conclusion

This patient’s late clinical presentation and systemic inflammation documented by blood tests, their response to anti-interleukin-1β treatment, the intermediate level of residual activity of mevalonate kinase, and its altered tertiary structure support that the novel c.1049A>C (Q350P) variant may contribute to an intermediate phenotype when combined in trans with another pathogenic *MVK* mutation. Consistent with the expanding spectrum of MKD, patients retaining 20%–60% residual mevalonate kinase activity may have milder inflammatory symptoms and a late onset compared to classic MKD. The identification of two MKD patients from a single center in 5 years suggests underestimation or underreporting of this potential cause of IS. Improved recognition of MKD among IS patients could lead to proper treatment with anti-interleukin-1β inhibiting agents and better outcomes. Larger epidemiological studies and multicenter efforts are needed to better define the clinical, biochemical, and genetic spectrum of MKD.

Take-home messages:

- MKD and other autoinflammatory diseases are rare causes of IS, including in adults.
- In patients with IS of undetermined etiology, markers of MKD include a positive family history, prior episodes of systemic inflammation, skin rash, and increased blood markers of inflammation that persist over time in the absence of another cause (example: vasculitis, infection, autoimmune disease, cancer).
- Mevalonic aciduria is consistent with MKD but can be missed if not measured during an inflammatory flare.
- Measurement of mevalonate kinase activity in lymphocytes showing low levels is consistent with MKD, but not widely available on a clinical basis.
- Identification by gene testing of biallelic pathogenic mutations establishes the diagnosis of MKD.

## Data Availability

The original contributions presented in the study are included in the article/supplementary material, further inquiries can be directed to the corresponding author/s.
